# The Cannabis Dilemma: A Review of Its Associated Risks and Clinical Efficacy

**DOI:** 10.1155/2015/707596

**Published:** 2015-10-11

**Authors:** Melvyn Weibin Zhang, Roger C. M. Ho

**Affiliations:** ^1^National Addiction Management Service, Institute of Mental Health, Singapore 539747; ^2^Department of Psychological Medicine, Yong Loo Lin School of Medicine, National University of Singapore, Singapore 119054

## Abstract

Cannabis, also known as marijuana, has 9-tetrahydrocannabinol as the main constituent. There has been strict legislation governing the utilization of cannabis locally and worldwide. However, there has been an increasing push to make cannabis legalized, in view of its potential medical and therapeutic effects, for various medical disorders ranging from development disorders to cancer treatment, and being an adjunctive medication for various neurological conditions. It is the aim of this review paper to explore the evidence base for its proposed therapeutic efficacy and to compare the evidence base supporting its proposed therapeutic efficacy with its known and well-researched medical and psychiatric side effects.

## 1. Introduction

Cannabis, also known as marijuana, has 9-tetrahydrocannabinol as the main constituent [1]. It is derived from an Indian hemp plant, commonly known as the cannabis salve. Cannabis is derived from the dried leaves of the plant. The leaves contain approximately 1–10% of marijuana and the residue contains approximately 8–15% of hashish [1]. A recent study which examined 10,597 high school seniors in the United States has estimated the overall prevalence of the abuse of hashish (which is a more potent form of cannabis) to be approximately 6.5% [2]. Of significance, 18.3% of those who have recently abused marijuana have also subsequently used hashish [2]. More recently, a study conducted by the United States Centre for Disease Control (CDC) [3] has reported that approximately 40.7% of individuals sampled have ever used cannabis, and approximately 23.4% of the individuals are still actively using it. Of significance, 8.6% of those who used cannabis are under the age of 13 years [3]. Locally, in Singapore, the prevalence of cannabis abuse has been increasing. In a recent report issued by the Central Narcotics Bureau, Singapore, [4] it has been reported that there has been a massive 29% increment in the number of individuals who have been arrested for cannabis abuse locally, when the statistics for the years of 2012 and 2013 are compared [4]. The most common method used to consume cannabis is via smoking the product, but it might also be consumed orally in various food products [5].

## 2. Pharmacology of Cannabis

Understanding the pharmacology of cannabis is essential as it helps to understand the side effects associated with cannabis and its proposed medical benefits. The main constituent that is responsible for the psychoactive properties is tetrahydrocannabinol [6]. In the central nervous system, there are 2 main subtypes of the cannabinoid receptors. These receptors are commonly known as cannabinoid-1 and cannabinoid-2 [7]. The CB1 receptors are most populated in the following regions: cortex, hippocampus, basal ganglia, cerebellum, and spinal cord [7]. In contrast, the CB2 receptors are most populated in those cells that are responsible for immune mediation [6]. Hence, the CB1 receptor has a consequential mediating effect on memory, cognition, and movement [1]. Both the CB1 and CB2 receptors are coupled to inhibitory G protein complexes, which via activation would cause an inhibition of adenylate cyclase. This in turn results in a decreased concentration of cAMP in the cell. Thus, activation would in turn result in the inhibition of neurotransmission [6].

Thus, when cannabis is consumed, the main ingredient which is tetrahydrocannabinol would bind to the CB1 receptors and this results in an activation of the G protein complexes. This would cause a resultant inhibition of the sodium channel, as well as the N and/Q type voltage gated channels. The inwardly rectifying potassium channels are stimulated instead [1, 6]. These changes lead to the euphoric feelings associated with its consumption [1, 6]. Apart from bringing about euphoric feeling, its utilization would also result in significant anticholinergic effects. The anticholinergic effects have been postulated to be responsible for the cognitive deficits arising from the consumption of cannabis. Other hormones, such as the luteinizing hormone, prolactin, and growth hormone production are also affected.

## 3. Clinical Symptoms Arising from Usage

For an individual who smokes cannabis, the typical peak effect would occur at approximately 30 minutes after the consumption. The individual might have the following clinical symptoms, which include anxiety or agitation, illusions, feelings of depersonalization, hallucinations, paranoid ideations, temporal slowing as well as impaired judgment, and attention. Other clinical signs of intoxication include red eyes, dryness of mouth, tachycardia, and increased appetite. These effects usually last for 2 to 4 hours. However, the effects of cannabis on cognition might last much longer, ranging from 5 to 12 hours. Those who consume cannabis orally might not experience such significant symptoms as the bioavailability is significantly reduced. Individuals who have consumed high doses might be acutely confused, and they might have other clinical symptoms such as hypotension and hypothermia as well as psychiatric manifestations such as psychosis.

## 4. Risks Associated with Usage of Cannabis

### 4.1. Psychiatric Manifestations Arising from the Usage of Cannabis

There are various psychiatric manifestations that might arise from the usage of cannabis. Anxiety disorders have been reported to be associated with cannabis usage. A recent meta-analysis [8] that looked at 31 studies involving 112,000 individuals has found a positive association between anxiety disorder and cannabis usage. It is postulated that individuals with underlying anxiety disorders might have used cannabis as a form of self-medication to help them cope [9]. It is also postulated that cannabis usage might predispose one towards an anxiety disorder due to the underlying genetic vulnerabilities, gender, and age [9]. In addition, there have been previous reports about the association between depressive disorders in cannabis users. Lev-Ran et al. [10] undertook a meta-analysis and examined 57 studies. It was reported that the odds for a heavy cannabis user to develop depressive disorder were 1.62. Hence, cannabis use has been associated with a modest increment in the risk for developing depressive disorder. There have been 2 proposed mechanisms that could account for this association. It might be possible that cannabis acting on the CB1 receptors in the brain could cause a regulation in the emotional experience [11]. It is also possible that cannabis use in itself is correlated with significant adverse life events that could enhance the likelihood of individuals acquiring depression [12]. Apart from cannabis and its association with depression, the usage of cannabis might also worsen both the severity and the duration of maniac symptoms in individuals who have an underlying diagnosis of bipolar disorder [13]. The proposed mechanism is that consumption of active ingredient in cannabis might result in changes in the dopaminergic responses [14]. This is supported by pharmacological and radiological imaging studies, which have found that there is dopaminergic hyperactivity in both psychosis and mania [13].

Apart from the association between cannabis usage and affective and anxiety disorders, it is commonly known that cannabis usage is associated with an increased incidence of psychosis. Cannabis usage might result in a twofold increment in the risk of acquiring schizophrenia and a corresponding fourfold increase in the risk of psychosis [15]. However, there are ethnic and genetic variants. It should be noted that individuals who are homozygous for the VAL/VAL alleles in the catechol-O-methyltransferase (COMT) genotype tend to be at enhanced risk. Those individuals who are homozygous for the MET/MET alleles in the COMT genotype tend to be not at enhanced risk [1]. In addition, the usage of cannabis has been linked with an earlier age of onset of psychosis [16]. Previous meta-analysis has reported that the average age of onset of psychosis for cannabis users was 2.70 years younger as compared to normal controls [16]. For individuals who are deemed to be clinically at high risk for acquiring psychosis, or who are prodromal, the results arising from meta-analysis have reported that there are more severe prodromal symptoms at baseline and an earlier onset of clinically high-risk symptoms [17]. Whilst there has been much research and evidence examining the association between cannabis usage and major mental disorders, a recent meta-analysis has found an association between cannabis use and personality traits [18]. It has been demonstrated that on-going cannabis usage is associated with much higher scores of global, positive, and disorganized schizotypal personality traits [18]. Of importance, current research has reported that cannabis usage is associated with an elevated risk of suicide in those with and without psychosis [19].

### 4.2. Medical Manifestations Arising from the Usage of Cannabis

The usage of cannabis is associated with multiple medical manifestations.  Table 1 summarizes some of the common medical complications associated with the usage of cannabis and the underlying pathophysiology.

More importantly, the usage of cannabis has particular implications on the development of the adolescent brain and hence has a mediating effect on cognitive processes [20]. Radiological studies, such as functional imaging studies, have revealed that there is a deficit in the neuropsychological performance of cannabis users [21]. The findings of the FMRI studies have revealed that there are lower activities on the right dorsolateral prefrontal and occipital cortices but more activation in the right posterior parietal cortex [22]. It has been postulated that chronic cannabis usage in adolescence is associated with less efficient executive functioning and attention, likely due to disruption on the frontoparietal connectivity, associated with a compensatory right prefrontal overactivity [23].

## 5. The Current Debate

The Federal Drug Enforcement Administration (DEA) has placed cannabis as a Schedule 1 drug and has considered cannabis to be one of the most dangerous substances available. In a statement previously released, the Federal Drug Enforcement Administration has reported that there is no currently accepted usage of cannabis for medical therapeutic purposes, as it has an inherent high risk for abuse [24, 25]. However, there has been a recent shift in the governmental process, as more states in America are approving what is termed as medical cannabis [24]. This is in view of the fact that more and more patients are believing that cannabis and its associated derivatives could help to relieve symptoms for conditions such as malignancy, retroviral disorders, and epilepsy. Medical or synthetic cannabis has been licensed overseas for medical use currently. Hence, there is an increasing push for the legalization of cannabis. A previous survey conducted by Charuvastra et al. (2005) [26] has reported that as many as 36% of individuals amongst a total sample of 960 participants have advocated for the legalization of cannabis. Hence, this in itself is a controversial issue. The legalization of cannabis, similar to how alcohol and tobacco have been legalized, might lead to more youth usage and abuse. The early years of experimenting with these drugs, such as cannabis, would lead to higher incidences of addiction and abuse. There will in turn be a higher rate of concurrent psychiatric disorders arising from the abuse of cannabis. Given this, it is important to understand the evidence base supporting the medical utilization of cannabis, given that it is commonly known that cannabis usage is in itself associated with multiple medical and psychiatric adverse effects and comorbid.

## 6. Medical Use of Cannabis

### 6.1. Pediatric Developmental and Behavioral Disorders

Medical cannabis has been proposed for usage in the treatment of pediatric developmental and behavioral disorders, such as autistic disorder. However, the best current evidence to date is limited to case series or single studies [27]. There has been a case study published reporting the utility of medical cannabis in the treatment of a 6-year-old boy with autism [28]. Medical cannabis has been reported to help improve hyperactivity, irritability, lethargy, and stereotyped behaviors for the child.

### 6.2. Cancer Treatment

Cannabis has been reported to be of utility in cancer treatment. There have been studies reporting the efficacy of medical synthetic cannabis in helping to inhibit cancer growth and the spread of cancer cells [29, 30]. Despite the promising initial findings, there have been conflicting results from other studies and this limits the generalization of the current findings.

### 6.3. Treatment for Neurological Conditions

Medical cannabis has been utilized as a form of treatment for epilepsy [31]. With regard to the evidence base pertaining to its clinical efficacy, thus far, there have only been 2 small double-blinded, placebo-controlled studies that have evaluated the clinical utility of this drug for treatment in epilepsy [32, 33]. Of significance, the results do vary significantly between the 2 studies, with one study showing that it is of benefit, whereas the other showed no improvement amongst the patients. Previous review studies did acknowledge that medical cannabis might be of therapeutic purpose for epilepsy, but the current evidence base is lacking [34].

Medical cannabis has been also recommended for the treatment of headache. There have been previous published reports of the utilization of cannabis for cluster headaches [35], but there is a lack of randomized controlled trials demonstrating the clinical efficacy of medical cannabis for the treatment of headaches [31]. Hence, it has been recommended that conventional treatments should be adequately tried first prior to the consideration of unconventional therapies. Conventional treatments include pharmacological medications and nonpharmacological treatment such as biofeedback, physical therapy, cognitive behavioral therapy, and injections such as nerve blocks [31].

For multiple sclerosis, nabiximols [31] has been looked into extensively and is currently available for treatment of multiple sclerosis associated spasticity in 11 countries. Individuals who have used the drug felt that there was as much as a 20% reduction in the symptoms that they have experienced. With regard to neuropathic pain, medical cannabis has demonstrated clinical efficacy [36, 37], but there have been limited studies conducted comparing its clinical efficacy to other conventional treatments. More importantly, the current trials have not rigorously evaluated the safety of using medical cannabis as a treatment for neuropathic pain. Utilization of medical cannabis for amyotrophic lateral sclerosis has been proposed, given that cannabis has been postulated to be able to help in the regulation of the immune system through its actions on the CB2 receptors. However, there remains a need for further research looking into this, to demonstrate the clinical benefits. For sleep related disorders, there remains a lack of current data validating the clinical effectiveness of cannabis. In particular, it has been highlighted that sleep problems might be experienced by individuals instead when cannabis is being withdrawn from their treatment [38].

### 6.4. Digestive Disorders

Medical cannabis has demonstrated clinical efficacy against dopamine antagonist for the treatment of chemotherapy induced nausea and vomiting [39]. However, there remains limited evidence comparing its antiemetic effects with other medications, such as the serotonin antagonists. There has been a lack of controlled studies thus far as well comparing its antiemetic effects [40]. For oncology patients, medical cannabis could also be used as an appetite stimulant. However, the results from current trials are mixed [41]. For patients with chronic inflammatory bowel disease, it is believed that medical cannabis could help in the reduction of inflammation. However, there remains a lack of objective evidence as there is no monitoring of endoscopic disease activity.

## 7. Illicit Use of Cannabis and Legislation in Singapore

In Singapore, cannabis has been included as a Schedule 1 drug in the 1961 Single Convention on Narcotic Drug [42]. Singapore is one of the countries amongst 184 countries to have signed this agreement to curb the cultivation of the plant. In Singapore, there are grave legal penalties associated with the abuse or possession of cannabis. Individuals are liable to be fined up to 20,000 Singapore dollars or be imprisoned for up to 10 years if they are found to have consumed or found to be in possession of the drugs [42]. The legal penalties for illegal trafficking of the drug are even more severe. Individuals will face the death sentence if they import or export cannabis of more than 500 grams, resin of more than 200 grams, or mixture of more than 1000 grams.

## 8. Evidence Supporting Usage

Although there have been numerous studies demonstrating the potential therapeutic efficacy of medical cannabis overseas, the results obtained should be interpreted with caution. The underlying reason for this is that most of the studies are limited to case reports or cross-sectional trials. There has not been comparison between medical cannabis and conventional treatment to determine the added benefits or the superiority of medical cannabis over conventional treatment. In addition, recent studies looking into the pharmacology of medical derived cannabis have yielded other concerning findings [43]. It should be noted that medical or synthetic cannabis tends to have higher affinities to the respective receptors. In particular, it has approximately 5 times higher affinity to CB1 receptors and 10 times higher affinity to CB2 receptors. These higher affinities are in turn likely to predispose individuals more to psychotic experiences. Forrester et al. [43] have reported a 5-fold increment in the rates of hallucinations as compared to conventional abused cannabis.

## 9. Conclusion

In conclusion, whilst there have been more evidences demonstrating the clinical efficacy of medical cannabis, these new evidences need to be interpreted with caution. There is a need for more randomized controlled trials demonstrating clearly the efficacy of medical cannabis for therapeutic purposes before widespread legalization takes place worldwide. There is still a need for there to be legalization in place, in order to avoid the abuse of “medical cannabis” in the same way that alcohol has been abused previously in view of the legislative changes.

## Figures and Tables

**Table 1:** 

Medical manifestations	Associated pathophysiology
Cannabis induced arteritis [44, 45], cannabis induced posterior circulation stroke [46], and myocardial infarction [47]	This is likely due to the vasoconstrictor effect of cannabis

Chronic cough, bullous emphysema, and chronic obstructive lung disorder [48, 49]	Cannabis smoke is known to contain polycyclic aromatic hydrocarbons and other carcinogens In addition, heavy use of cannabis is likely to lead to generalized airway inflammation

Cannabis hyperemesis syndrome [50]	This seems to be a paradoxical effect associated with the usage of cannabis

Adipose tissue insulin resistance, pancreatitis [51, 52]	Proven underlying mechanisms have not been ascertained
